# Effect of Multimodal Interventions on Pain, Stress, and Neurodevelopmental Outcomes in Neonates With Malignant and Benign Tumors: Protocol for a Systematic Review

**DOI:** 10.2196/77969

**Published:** 2026-01-13

**Authors:** Sharath Hullumani, Shrutika Sharad, Aishik Ghosh, Rutuja Sawalkar, Irshad Qureshi

**Affiliations:** 1 Faculty of Physiotherapy, Datta Meghe Institute of Higher Education and Research (DU), Sawangi Meghe, Wardha, Maharashtra, 442001, India, 91 9964066927

**Keywords:** stress reduction, non-pharmacological therapy, integrative therapy, pain, newborns, neonate

## Abstract

**Background:**

Both benign and malignant tumors in neonates frequently necessitate invasive diagnostic and therapeutic procedures, exposing infants to significant pain and stress during critical periods of brain development. Procedural stress can disrupt the hypothalamic-pituitary-adrenal axis, affect synaptic pruning, and alter myelination, potentially leading to long-term cognitive and neurodevelopmental impairments. While standard medical care remains the cornerstone of management, there is growing interest in multimodal interventions—including integrative therapies, physiotherapy, and non-pharmacological approaches such as massage, music therapy, kangaroo care, and sensory stimulation—to mitigate discomfort and support neurodevelopmental outcomes.

**Objective:**

This study aimed to systematically evaluate how well multimodal therapies reduce stress and suffering while enhancing neurodevelopmental outcomes in infants with both benign and malignant tumors.

**Methods:**

The PRISMA-P (Preferred Reporting Items for Systematic Review and Meta-Analysis Protocols) standards will be followed in the conduct of this systematic review. Studies that use multimodal therapies (eg, physiotherapy, massage, music therapy, kangaroo care, or other non-pharmacologic techniques) for newborns with proven malignant or benign tumors will be considered. A thorough literature search will be conducted using databases such as the PubMed, Embase, and CINAHL, Cochrane, and CancerLi. Cohort studies, case-control studies, quasi-experimental studies, and randomized controlled trials will all be considered. Measures of pain (such as validated neonatal pain scales), stress (such as cortisol levels and behavioral markers), and neurodevelopment (such as the Bayley Scales of Infant Development) will be the main objectives.

**Results:**

The anticipated timeline for completing this systematic review is June 2025 to December 2027. Two independent reviewers will conduct each stage of the review process in a blinded manner to minimize bias.

**Conclusions:**

This systematic review aims to provide evidence-based insights into the role of multimodal interventions in enhancing standard care for neonates undergoing tumor-related procedures. Conclusions regarding clinical impact will be conditional on the quality and consistency of the evidence, as assessed using GRADE (Grading of Recommendations, Assessment, Development, and Evaluation). The findings are expected to guide future research, inform cautious clinical recommendations, and support the development of integrative strategies to optimize neurodevelopmental outcomes in this vulnerable population.

## Introduction

### Rationale

Newborns with both benign and malignant tumors frequently undergo invasive diagnostic and therapeutic procedures—including surgery, chemotherapy, and prolonged hospital stays—subjecting them to substantial physical and psychological stress. Early-life stress can adversely affect behavior, brain development, and long-term health outcomes by disrupting the hypothalamic-pituitary-adrenal axis, impairing synaptic pruning, and altering myelination. Non-pharmacological and physiotherapeutic interventions, such as music therapy, massage, kangaroo care, and sensory stimulation, are increasingly being investigated as strategies to mitigate these negative effects. However, the efficacy of such multimodal approaches in neonatal oncology remains poorly supported by aggregated evidence [1-3].

The rarity and heterogeneity of neonatal tumors make it challenging to define their true prevalence, sites of origin, and clinical behavior. Neonatal tumors occur in approximately 1 per 12,500‐27,500 live births and account for roughly 2% of pediatric malignancies. Despite their low incidence, these tumors offer a unique opportunity to study tumor biology with minimal confounding from environmental factors. Common tumor types include teratomas, neuroblastomas, mesoblastic nephromas, and fibromatoses, which often present as a mass at birth and are frequently detected through prenatal ultrasonography [4].

Although benign tumors may occasionally undergo malignant transformation, true malignancies in the neonatal period are rare. Histological features do not always correlate with clinical behavior, complicating accurate classification. Benign tumors can be life-threatening depending on their size or location, whereas some histologically malignant tumors may exhibit indolent or ambiguous behavior [5-11]. Genetic predispositions, such as constitutional chromosomal abnormalities, can favor tumor development during the fetal and neonatal stages. Environmental exposures—including ionizing radiation, maternal medications, infections, and maternal tumors—may also contribute to tumor risk, although evidence varies in strength [6]. Given these complexities, a systematic synthesis of evidence on multimodal, non-pharmacological interventions is critical to inform supportive care strategies aimed at minimizing procedural stress and optimizing neurodevelopment in this vulnerable population.

### Objective

This study aimed to systematically evaluate the efficacy of multimodal, non-pharmacological interventions—including physiotherapy, integrative therapies, and sensory-based approaches—in reducing procedural pain and stress, and enhancing short- and long-term neurodevelopmental outcomes in neonates with benign and malignant tumors. The review will also identify intervention timing, delivery methods, and gaps in current evidence to guide future research and clinical practice.

## Methods

### Overview

The PRISMA-P (Preferred Reporting Items for Systematic Reviews and Meta-Analyses Protocols) guidelines will be followed in conducting this review. Evidence from observational and interventional trials that evaluate the use of multimodal (non-pharmacological) treatments in newborns with tumors will be identified and synthesized in this review. A narrative synthesis and meta-analysis (if applicable) will be performed to evaluate findings and guide future research and clinical practices.

### Eligibility Criteria

The eligibility criteria were determined using the PICOT (population, intervention, comparison, outcome measures, and timing) framework

The population (P) included neonates (≤28 days) diagnosed with benign or malignant tumors who were treated with non-pharmacologic interventions.

The intervention (I) was multimodal, non-pharmacological interventions, including but not limited to physiotherapy (eg, chest physiotherapy or gentle handling), sensory stimulation (visual, auditory, or tactile), music therapy, massage therapy, Kangaroo mother care, non-nutritive sucking, and additional integrative or supportive non-pharmacological techniques.

The comparison (C) was between standard medical care without adjunct non-pharmacologic intervention and the absence of intervention or placebo controls, where applicable.

The outcome measure (O) was pain alleviation; this was assessed using validated neonatal pain scales, such as the Premature Infant Pain Profile (PIPP) orPremature Infant Pain Profile-Revised (PIPP-R) and Neonatal Infant Pain Scale (NIPS).

The main aim was stress reduction that was measured via physiological markers (eg, salivary or serum cortisol) and behavioral indicators (eg, facial expressions, crying, and agitation); this assessment was performed immediately or short-term, as indicated above.

Neurodevelopmental outcomes were assessed using standardized tools such as the Bayley Scale of Infant and Toddler Development, electroencephalography, and relevant imaging markers. Short-term (up to 1 mo post-intervention) and long-term follow-up (≥1 mo to assess ongoing neurodevelopment) assessments were performed.

Subgroup analyses are pre-specified for the (1) intervention modality, (2) tumor type (benign vs malignant), and (3) outcome category. This structure minimizes uninformative averaging.

### Study Design

The study will be focused on observational studies, experimental studies, and randomized controlled trials (RCTs).

### Languages of the Included Studies

Only studies published in English or with available translations will be included. This restriction is due to resource limitations for accurate translation and verification. To minimize potential bias, non-English abstracts will be screened, and studies deemed highly relevant will be noted, with attempts to access translations when feasible.

### Publication Date

Studies published from January 2000 to May 2025 will be included. No restrictions were applied on the year of publication to ensure the inclusivity of all relevant evidence.

### Publication Type Included

The types of studies included were both published articles and gray literature.

### Information Sources

A comprehensive search will be conducted using both electronic databases and manual methods to identify studies relevant to multimodal, non-pharmacological interventions in neonates with tumors. The electronic databases searched included PubMed, Embase, Cochrane Library, CancerLit (an oncology-specific database), and Google Scholar (to supplement formal database searches). Search strategies will combine controlled vocabulary terms (eg, MeSH and Emtree) and free-text keywords, customized for each database. Tumor-specific terms (eg, neonatal tumor, neoplasm, neuroblastoma, teratoma, and germ cell tumor), intervention terms (eg, physiotherapy, massage, music therapy, and kangaroo care), and validated neonatal outcome measures (eg, PIPP-R and NIPS) will be included. The full database-specific search strings are provided in Checklist 1.

### Eligibility of Evidence

Only studies available in full text will be included. Case reports, case series, and expert opinions will be excluded from data extraction due to limited generalizability. Both published literature and gray literature (theses, conference abstracts, clinical trial registries) will be screened, provided full text is accessible.

### Search Strategy

The search strategy will combine controlled vocabulary terms (eg, MeSH and Emtree) with free-text keywords, tailored for each database. Key terms will include “physiotherapy,” “stress reduction,” “non-pharmacological therapy,” “integrative therapy,” “pain,” and “newborn,” along with tumor-specific terms (ie, “neonatal tumor,” “neoplasm,” “neuroblastoma,” and “teratoma”). Truncation (*) and Boolean operators (AND, OR) will be used to ensure comprehensive retrieval. Medline and the Cochrane Library will particularly utilize MeSH terms for additional coverage. The full database-specific search strings are provided in Checklist 1. To ensure the inclusion of the most recent studies, all searches will be updated prior to the final analysis. In addition, trial registries, investigator contacts, and conference proceedings will be searched for unpublished studies. Both published and gray literature (eg, conference abstracts, theses, and clinical trial registries) will be included if the full text is available.

### Study Selection and Synthesis

Observational studies without control groups will be included in narrative synthesis but excluded from quantitative meta-analysis. Only studies in English or with available translations will be included, with non-English abstracts screened for potential relevance.

### Selection Process and Data Collection Process

Two calibrated and independent reviewers (SH and SS) will perform the study in two stages. The first step involves screening abstracts and titles for alignment with the inclusion criteria and research question. To decide on the final inclusion, the full texts of possibly pertinent research are examined in the second stage. This procedure will be guided by the inclusion and exclusion criteria. In phase 1, the titles and abstracts of the chosen papers will be read by the two reviewers, SH and SS. The entire texts of the previously included papers will be read by the same reviewers (SH and SQ) in phase two. The selection criteria will be the same as those from the first round. A third reviewer (IQ) will make the decision if there is any dispute.

### Data Items

The data synthesis will concentrate on assessing how well multimodal therapies work to improve neurodevelopmental outcomes, pain, and stress in newborns with benign and malignant tumors. Pre-established outcome measures will be used to aggregate the quantitative and qualitative data from qualified research. A narrative summary will be presented for each of the included studies, organized according to the demographic characteristics, intervention type, and outcomes that were measured.

A meta-analysis will be carried out using R software (version 4.1.2; R Foundation for Statistical Computing), including the tidyverse package (version 1.3.2) when the outcome data are sufficiently consistent across trials in terms of the design, intervention, and measurement instruments. For continuous outcomes, such as pain scores, cortisol levels, and neurodevelopmental test scores, effect sizes will be presented as 95% CI or the mean difference or standardized mean difference. The risk ratio or odds ratio with 95% CI will be used to express dichotomous outcomes, such as the presence or absence of adverse occurrences. Given the expected clinical and methodological heterogeneity, a random-effects model will be used.

### Assessment of Risk of Bias in Included Studies

The risk of bias (RoB) in included studies will be assessed independently by two reviewers (SH and SS), with disagreements resolved by a third reviewer (AG). The choice of the RoB tool will depend on the study design: for RCTs, the RoB 2.0 will be used; for non-randomized intervention studies, ROBINS-I will be used; and for observational studies, ROBINS-E will be used [12].

Before formal assessment, reviewers will discuss each tool and standardize the evaluation criteria, followed by a calibration exercise on a sample of included studies to ensure consistency. Inter-rater agreement will be calculated using Cohen κ, with κ≥0.80 considered acceptable. The RoB results will be presented according to each tool’s recommended format and visualized using Robvis [13]. Publication bias will be assessed if ≥10 studies are available for a given outcome. Funnel plots will be generated, and asymmetry will be tested using the Egger or Begg tests, as appropriate. This structured approach ensures reproducibility, transparency, and reliable evaluation of the methodological quality across diverse study designs.

### Data Synthesis

Data synthesis will be conducted according to study design, with RCTs pooled using RoB 2 assessments, non-randomized intervention studies evaluated with ROBINS-I, and observational exposure-based studies assessed using ROBINS-E. Where sufficient homogeneous RCT data are available, meta-analyses will be performed using standardized mean differences for continuous outcomes and relative risks for dichotomous outcomes, each reported with 95% CIs. Funnel plots and Egger tests will be applied only to homogeneous RCT subsets to assess publication bias. Given the anticipated heterogeneity across interventions, tumor types, and outcome measures, a narrative synthesis will serve as the primary approach. This narrative summary will describe key outcomes, intervention types, and their effects on pain, stress, and neurodevelopment; highlight patterns and gaps in the evidence; and include pictorial representations such as summary tables and figures to improve clarity. Subgroup analyses will be conducted, where possible, to explore variations in intervention effects according to participant characteristics (eg, tumor type, gestational age, and baseline clinical status) and intervention attributes (eg, modality, frequency, and timing). This combined quantitative and qualitative approach ensures a rigorous and comprehensive synthesis of available evidence while accounting for heterogeneity and supporting meaningful conclusions regarding multimodal interventions in neonates with tumors.

### Confidence in Cumulative Evidence

Using the GRADE (Grading of Recommendations, Assessment, Development, and Evaluation) method, this systematic review will be assessed. When it comes to handling neonates with respiratory distress syndrome, the GRADE approach may be able to perform a thorough evaluation of the presence of evidence that supports the efficacy of multimodal treatments.

## Results

The anticipated timeline for completing this systematic review is June 2025 to December 2027. Two independent reviewers will conduct each stage of the review process in a blinded manner to minimize bias. Regular team meetings will be held to resolve any discrepancies or conflicts, with all discussions documented to ensure transparency and reproducibility. The review will be carried out in strict adherence to the PRISMA-P (Preferred Reporting Items for Systematic Review and Meta-Analysis Protocols) guidelines, and the protocol has been prospectively registered with PROSPERO (CRD420251053012). Upon completion, the full review and its findings will be prepared and submitted for peer-reviewed publication.

## Discussion

### Anticipated Findings

It is anticipated that this systematic review will identify a spectrum of non-pharmacological interventions capable of reducing pain and physiological stress while promoting early neurodevelopment in neonates diagnosed with benign or malignant tumors. The evidence may highlight specific multimodal strategies—such as physiotherapy, sensory stimulation, and integrative therapies—that are both effective and feasible within neonatal oncology and critical care contexts. These findings are expected to contribute valuable insights into how supportive, non-pharmacological approaches can complement standard medical treatments in this highly vulnerable population.

### Strengths and Limitations

This protocol has been developed in alignment with the MOOSE (Meta-Analyses and Systematic Reviews of Observational Studies) and PRISMA-P guidelines, ensuring methodological rigor and transparency. Two independent reviewers will screen and extract data, with a third reviewer mediating any disagreements, thereby minimizing selection and extraction bias. However, certain limitations are anticipated. Restricting inclusion to English-language studies may reduce the comprehensiveness of the evidence base due to translation constraints. Additionally, the inclusion of non-randomized studies may introduce bias; however, this will be systematically assessed and discussed as part of the review’s limitations. Despite these constraints, the review will help identify knowledge gaps and inform priorities for future empirical and interventional research in neonatal oncology care.

### Conclusion and Dissemination

By synthesizing available evidence on multimodal non-pharmacological therapies, this review aims to clarify their role in enhancing both clinical and developmental outcomes in neonates with tumors. The findings will contribute to the formulation of evidence-based recommendations and integrated care strategies that prioritize comfort, neuroprotection, and developmental support. Dissemination of the results will occur through peer-reviewed publication, presentations at neonatal and pediatric conferences, and sharing within professional clinical and academic networks to ensure the translation of findings into practice and guide future research directions.

## Supplementary material

10.2196/77969 Checklist 1 PRISMA checklist.

## Abbreviations

GRADE: Grading of Recommendations, Assessment, Development, and Evaluation
MOOSE: Meta-Analyses and Systematic Reviews of Observational Studies
NIPS: Neonatal Infant Pain Scale
PICOT: population, intervention, comparison, outcome measures, and timing
PIPP/PIPP-R: Premature Infant Pain Profile
PRISMA-P: Preferred Reporting Items for Systematic Review and Meta-Analysis Protocols
RCT: randomized controlled trial
RoB: risk of bias
